# Deep learning system for automated detection of posterior ligamentous complex injury in patients with thoracolumbar fracture on MRI

**DOI:** 10.1038/s41598-023-46208-7

**Published:** 2023-11-03

**Authors:** Sang Won Jo, Eun Kyung Khil, Kyoung Yeon Lee, Il Choi, Yu Sung Yoon, Jang Gyu Cha, Jae Hyeok Lee, Hyunggi Kim, Sun Yeop Lee

**Affiliations:** 1 Department of Radiology, Hallym University Dongtan Sacred Heart Hospital, 7, Keunjaebong-gil, Hwaseong-si, Republic of Korea; 2 Department of Radiology, Fastbone Orthopedic Hospital, Hwaseong-si, Republic of Korea; 3 Department of Neurologic Surgery, Hallym University Dongtan Sacred Heart Hospital, Hwaseong-si, Republic of Korea; 4 Department of Radiology, Soonchunhyang University Bucheon Hospital, Bucheon, Republic of Korea; 5 Department of Radiology, Kyungpook National University Hospital, Daegu, Republic of Korea; 6 DEEPNOID Inc., Seoul, Republic of Korea

**Keywords:** Diseases, Trauma, Musculoskeletal system, Bone, Medical imaging, Bone imaging, Magnetic resonance imaging

## Abstract

This study aimed to develop a deep learning (DL) algorithm for automated detection and localization of posterior ligamentous complex (PLC) injury in patients with acute thoracolumbar (TL) fracture on magnetic resonance imaging (MRI) and evaluate its diagnostic performance. In this retrospective multicenter study, using midline sagittal T2-weighted image with fracture (± PLC injury), a training dataset and internal and external validation sets of 300, 100, and 100 patients, were constructed with equal numbers of injured and normal PLCs. The DL algorithm was developed through two steps (Attention U-net and Inception-ResNet-V2). We evaluate the diagnostic performance for PLC injury between the DL algorithm and radiologists with different levels of experience. The area under the curves (AUCs) generated by the DL algorithm were 0.928, 0.916 for internal and external validations, and by two radiologists for observer performance test were 0.930, 0.830, respectively. Although no significant difference was found in diagnosing PLC injury between the DL algorithm and radiologists, the DL algorithm exhibited a trend of higher AUC than the radiology trainee. Notably, the radiology trainee's diagnostic performance significantly improved with DL algorithm assistance. Therefore, the DL algorithm exhibited high diagnostic performance in detecting PLC injuries in acute TL fractures.

## Introduction

Thoracic and lumbar (TL) spine fractures are among the most common fractures, and not only the fracture morphology but also the posterior ligamentous complex (PLC) injury is considered to be very important in determining the patient's treatment^1,2^. In a previous study, it was reported that approximately 53% of acute TL spine trauma cases were accompanied by PLC injury^3^. TL fractures are often evaluated through the Thoracolumbar Injury Classification and Severity Score (TLICS), which includes PLC injury evaluation^2,4^. The PLC is a posterior structure that affects the stability of the vertebral fracture site and is composed of the supraspinous and interspinous ligaments, ligamentum flavum, and facet joint capsule^4,5^. Magnetic resonance imagining (MRI) has become the most frequently used modality due to its high sensitivity and accuracy in detecting PLC injuries^4–6^. On MRI, PLC injuries are best assessed in T2-weighted fat saturation sagittal scans^5^, with injury diagnosis based on the presence of disruption in structures showing low signal intensity (SI) or suspected injury-related high SI changes^5,7,8^. TLICS categorizes PLC injuries into intact, indeterminate, or injured based on the severity of these findings. However, it's important to note that high SI changes can include false positives, such as edema, leading to reports of high sensitivity but low specificity in MRI evaluations^8,9^. As a result, the radiologist's judgment in MRI-based PLC injury assessment is crucial and can significantly influence surgical decisions, as TLICS plays a pivotal role in determining whether a patient should undergo surgical treatment^4^.

The development of artificial intelligence (AI) technology has led to an increasing number of AI decision support systems to help to prevent or reduce radiologists burnout due to abundant imaging volume^10–13^. Deep learning (DL) technology has been applied by various studies in the radiologic diagnosis of TL fractures^10,12–17^. However, most of these used DL to detect TL fractures in radiography and computed tomography (CT) and their usefulness has been proven for segmentation and detection of fractures. However, few studies for DL of TL fractures are based on MRI. Specifically, no MRI-based DL study has focused on PLC injury of multiple anatomical structures.

Therefore, in this study, we aim to develop a DL algorithm for automated detection and localization of PLC injury in TL fractures on MRI and evaluate its diagnostic performance.

## Methods

This study was reviewed after obtaining Institutional Review Board (IRB) approval from each human research ethics committee of two institutions (Hallym University Dongtan Sacred Heart Hospital IRB and Soonchunhyang University Bucheon Hospital IRB). The requirement for written informed consent from the study subjects was waived by the IRB of Hallym University Dontan Sacred Heart Hospital and Soonchunhyang University Bucheon Hospital due to the retrospective study design. All the methods of this study were performed in accordance with the relevant guidelines and regulations.

### Subjects (Supplementary Method 1)

**Figure 1:**
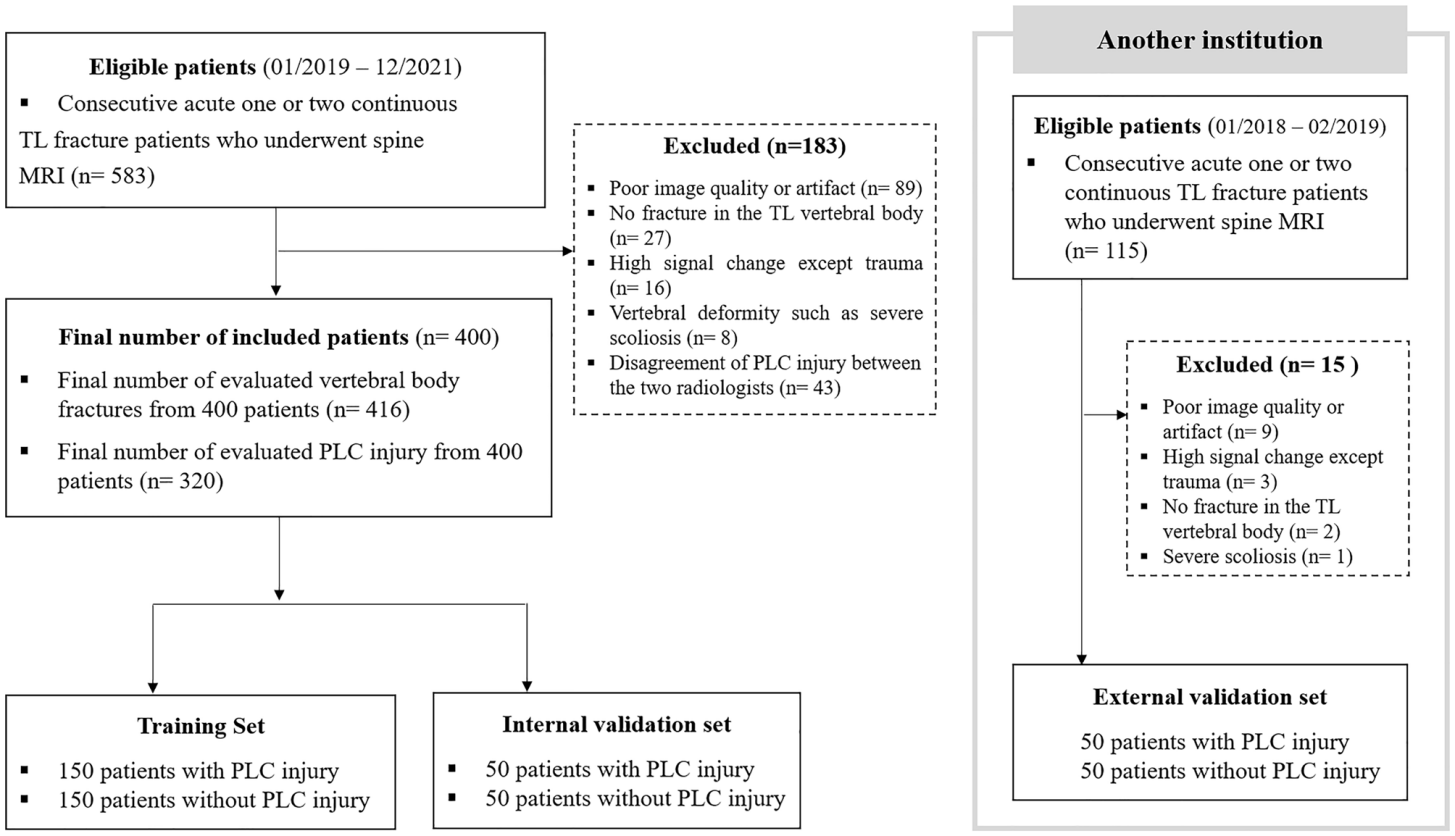
Flow chart for selection of the study population (PLC, posterior ligamentous complex; TL, thoracolumbar).

Between January 2019 to December 2021, the image database contained 583 consecutive patients who underwent baseline thoracic or lumbar spine MRI after trauma and were diagnosing them with one or two continuous acute TL fractures. Exclusions were made based on specific criteria as shown in Figure 1. Ultimately, we included 400 patients with acute TL fractures, randomly divided into two groups: a training dataset of 300 patients (150 with PLC injury and 150 with normal PLC) and an internal validation dataset of 100 patients (50 with PLC injury and 50 with normal PLC). For external validation, we collected data from a different institution using different MRI scanners. The exclusion criteria of both external and internal validation datasets were identical, and 15 patients were excluded. Ultimately, the external validation set consisted of 50 TL fracture patients with PLC injury and another 50 TL fracture patients with normal PLC. Table 1 summarizes the demographic information of the patients.

**Table 1:** Clinical characteristics of training and validation datasets in this study.

	Training set—injured PLC (n = 150)	Training set—normal PLC (n = 150)	Internal validation set—injured PLC (n = 50)	Internal validation set—normal PLC (n = 50)	External validation set—injured PLC (n = 50)	External validation set—normal PLC (n = 50)
Age	66.13 ± 14.97	63.13 ± 17.89	68.26 ± 13.49	61.60 ± 16.94	57.34 ± 19.85	64.66 ± 16.45
Sex (male/female)	65/85	38/112	19/31	12/38	26/24	14/36
Location of fracture						
Only T	52	68	15	26	15	14
Only L	98	80	34	24	32	34
T12 and L1	0	2	1	0	3	2
Number of fracture						
1	144	144	48	48	37	46
2	6	6	2	2	13	4
Total number of fracture	156	156	52	52	63	54
Number of injured PLC						
1	62	NA	22	NA	32	NA
2	86	NA	24	NA	16	NA
3	2	NA	2	NA	2	NA
TLICS score	4.22 ± 0.81	1.78 ± 0.42	4.30 ± 0.80	1.82 ± 0.39	4.29 ± 0.89	1.59 ± 0.50

^†^PLC, posterior ligamentous complex; T, thoracic; L, lumbar, TLICS, Thoracolumbar Injury Classification and Severity Score; NA, Not applicable.

### Image acquisition

For the training and internal validation sets, all MRI scans, which included routine thoracic or lumbar spine MRI, were conducted with a 3.0 T unit (Skyra 3.0 T, Siemens, Erlangen, Germany) in this institution. For external validation set, 1.5 T MRI (Signa HDxt 1.5 T, GE Healthcare, Milwaukee, Wis, USA) was used. All spine MRI sequences included fat suppression (FS) using the Dixon technique T2-weighted image (WI) in the sagittal plane for trauma evaluation. And we used only one midline image of sagittal FS-T2WI for the training, internal and external validation sets. The specific spine MRI parameters of all scans in all datasets are presented in Supplementary Tables 1, 2.

### Data collection and annotation

Spine MR images of training and test sets were reviewed by two musculoskeletal (MSK) radiologists with nine and 10 years of experience, respectively. Manual segmentation for each fractured vertebral body, background soft tissue anatomy (BA) including PLC above and below fractured vertebral body level, and injured PLC on sagittal FS-T2-WI was performed by one MSK radiologist with 10 years of experience using in-house software (DEEP:LABEL, DEEPNOID) (Supplementary Fig. 1).

### DL algorithm development

**Figure 2:**
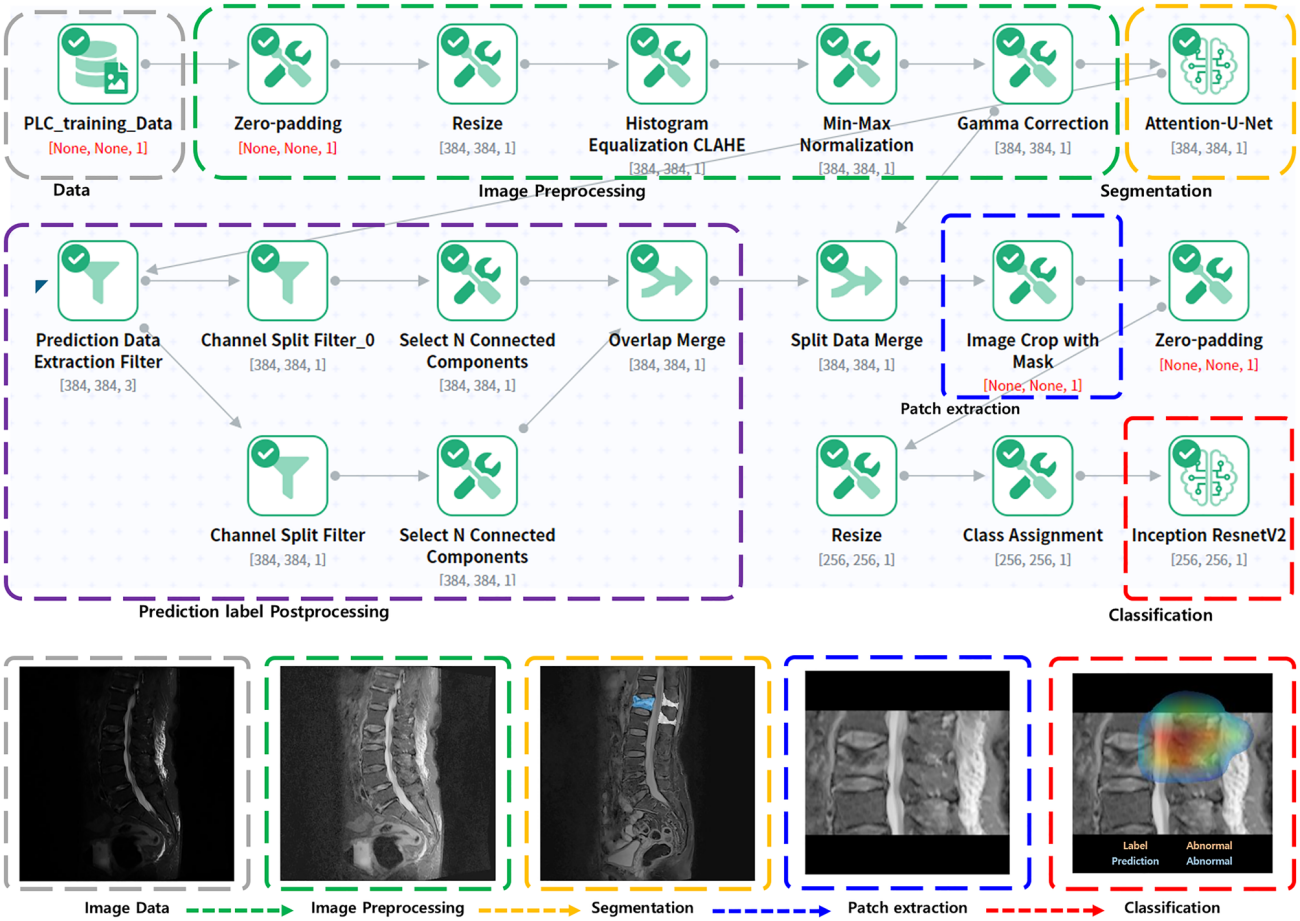
The process of deep learning algorithm system, which consists of the two algorithms Attention U-net and Inception-ResNet-V2. Imaging preprocessing was performed by zero padding, resize, histogram equalization, Min–Max normalization, and gamma correction. After segmentation of fractured vertebral body and background soft tissue anatomy by trained Attention U-net, post-processing of predicted segmentation (label) and patch extraction were performed. Final classification task was performed by Inception-ResNet-V2.

In this study, the proposed DL system consisted of a two-step process after preprocessing of the input image: (1) segmentation and patch extraction, and (2) classification. To ensure that the DL model can perform segmentation and classification more efficiently, we performed image preprocessing in the order shown in the green box in Fig. 2. To preserve the proportions of the image, we performed zero padding and resized it to 384 × 384 (height x width). Thereafter, contrast was enhanced, and brightness was adjusted as a preprocessing to improve the automatic segmentation performance of the fractured vertebral bodies. In MRI images, the spine has specific features, such as constant shape and pixel value, but to find a vertebral body with an acute fracture, we need to find changes in image features, such as bone marrow edema. Therefore, the contrast of pixel values is an essential factor in determining whether a vertebral body has an acute fracture, and to improve it, we used the contrast limited adaptive histogram equalization (CLAHE) and the enhancement technique of gamma correction^18,19^. Min–Max Normalization was also applied during the preprocessing, and the preprocessed image described above became the input to the first DL architecture, and the next step, the segmentation task, was performed.

#### Segmentation and patch extraction

**Figure 3:**
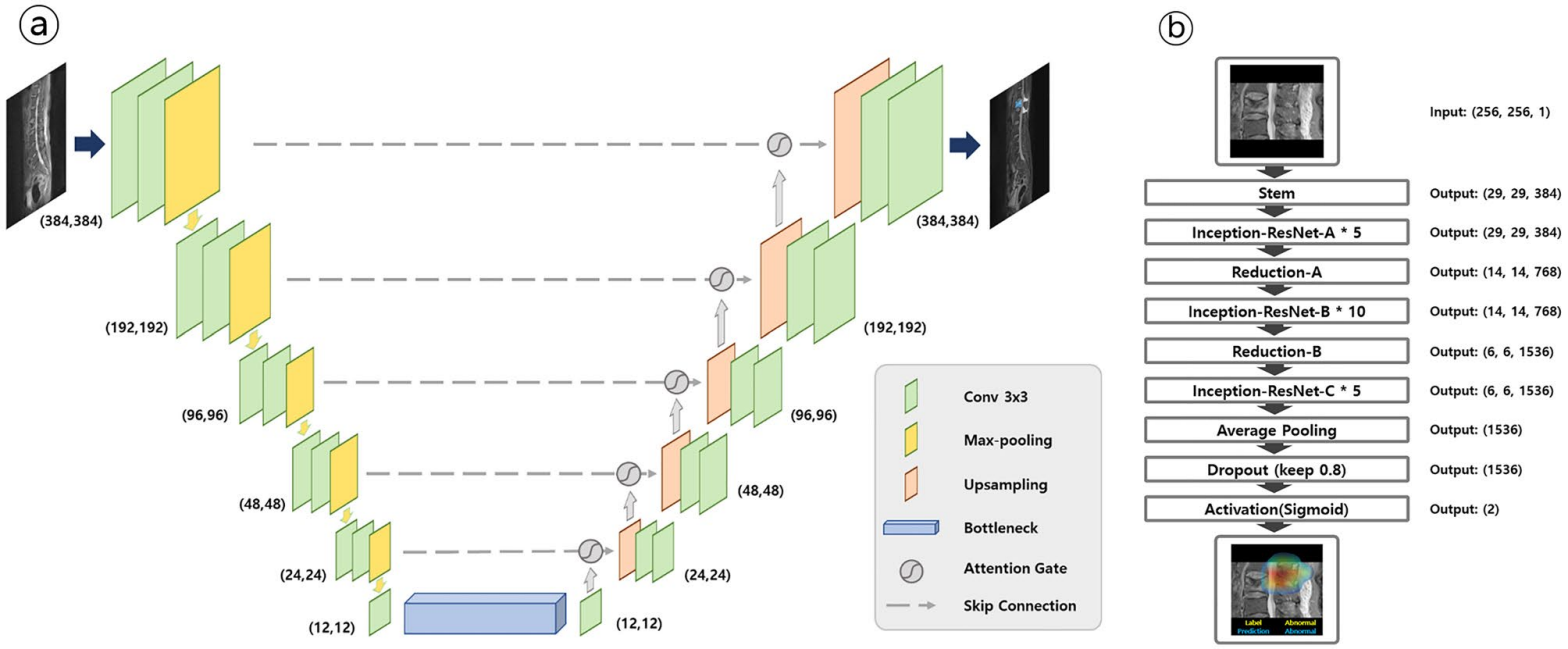
Schematic architectures of the first step deep learning algorithm (Attention U-net) and second step deep learning algorithm (Inception-ResNet-V2). **(a)** A block diagram of the Attention U-Net segmentation model in this study. The input image is gradually filtered and down-sampled at each step in the encoding portion of the network. In addition to the basic structure of U-Net, where Encoder for obtaining overall context information of the image and Decoder for accurate localization are symmetrically configured, the accuracy is improved by emphasizing only the necessary features using Attention Gates (AGs) for each skip connection. **(b)** The overall scheme of the Inception-ResNet-v2 networks in this study. As a first step, it goes through a Stem block with a general Convolution and Pooling structure. In the second step, it goes through a combination of Inception-ResNet Block, which combines Inception's features and ResNet's strengths, and Reduction Block, which generates size changes in features. Finally, the probability value of the class is extracted by making the feature into a one-dimensional vector through Global Average Pooling.

The DL architecture for segmenting acute vertebral body fracture and BA through preprocessed input images is shown in the yellow box in Fig. 2, and the detailed structure is shown in Fig. 3A. The DL algorithm used for training is Attention U-Net, which applies an attention mechanism to increase the concentration of features based on U-Net, the most commonly used segmentation algorithm in medical image segmentation^20^. This architecture applies attention gates to each skip connection in the decoder part of U-Net to emphasize the target feature, which improves segmentation performance. The following parameters were used for attention U-net: 200 epochs; batch size of 32; dropout rate of 0.05; group normalization with group size of 16; RMS Prop optimizer; learning rate of 0.0025; and learning rate decay of 0.99 every training step.

Post-processing of the predicted labels extracted through the segmentation task is performed in the order described in the purple box in Fig. 2. This post-processing is intended to reduce noise by removing all but the largest components of the TL fracture and BA predicted by the segmentation algorithm (Attention U-net), and utilizes the most reliably segmented components to extract patches as represented by the blue box in Fig. 2. This patch extraction was performed by cropping the image with a slight margin based on the location of the determined component. In the case of images where the fractured vertebral body and BA were not predicted at all, the entire image was processed as a patch. This final patch was then used as an input to the second DL architecture to perform the classification task of whether the PLC was injured or not.

#### Classification

This final extracted patch was also preprocessed to 256 × 256 by zero-padding and resizing as the original image, and the DL architecture that utilizes it as an input image to classify PLC injury is shown in the red box in Fig. 2, and the detailed structure is shown in Fig. 3B. The DL algorithm we used for training was Inception-ResNet-V2^21^, which combines the strengths of Inception and ResNet by applying the concept of residual connections to the Inception V4 architecture. The outcome of the model is a binary result of whether the PLC is injured, and we used Gradient-weighted class activation mapping (Grad-Cam) to visualize in a heatmap which parts of the image the model used to make its judgment. The following parameters were used: 200 epochs; batch size of 16; dropout rate of 0.05; Adam optimizer; learning rate of 0.0001; and learning rate decay of 0.99 every training step (Fig. 3B). Additionally, we used data augmentation such as 15º rotation and gamma contrast (0.7–1.7). Because the injured PLC was small and due to the anatomical characteristics of these patches, other augmentation operations that included interpolation, flipping, and mirroring were not performed. The performance of the DL algorithm was investigated using internal and external validation datasets.

### Comparison of the proposed DL system (two serial DL algorithms) with other DL algorithms.

To verify the superiority of the DL system proposed in this study, we conducted several comparative experiments on an external validation dataset. Through these comparative experiments, we can compare the performance of the backbone DL architecture used in each step with various other DL algorithms and explain the rationales for the backbone DL architecture settings of the proposed DL algorithm system. Furthermore, we eliminated two processes, segmentation task and patch extraction, and compared the PLC injury diagnosis performance of the classification algorithms on the original image (single classification algorithm result) with that of the proposed DL system (two serial DL algorithms) to validate the usefulness of the two processes.

### Observer performance test

We conducted an observer performance test to investigate the diagnostic performances between the DL algorithm and radiologists. Two newly invited radiologists independently reviewed the images of the test set (the first session). One radiologist was an MSK radiologist (reader 1[R1]) had ten years of experience in radiology, including musculoskeletal radiology. Another radiologist was a trainee (reader 2[R2]) who had three years of experience in radiology, including MSK radiology. We did not involve the radiologists who defined the reference standard for the external validation dataset to participate in this test. Additionally, if there is a radiologist whose diagnostic performance is significantly lower than that of the DL algorithm or other radiologist, we evaluated whether the DL algorithm could improve the diagnostic performance of the radiologists after one-month washout period (the second session).

### Statistical analysis

The sensitivity, specificity, positive predictive value (PPV), negative predictive value (NPV) and accuracy of the DL algorithm and radiologists were analyzed. The area under the curve of the receiver operating characteristics (AUROC) analyses were also performed to compute the discriminating power of the DL algorithm and the two radiologists. To analyze the diagnostic performance between the DL algorithm and the reviewers, we compared the area under the curve using the Delong’s test. McNemar’s test was performed to compare the sensitivity and specificity of the DL algorithm with those of each of the two radiologists. McNemar's test was also used to compare the sensitivity and specificity of the radiologist with DL algorithm assistance and the radiologist without DL algorithm assistance. Statistical analyses were performed using R statistical software version 4.0.3 and MedCalc version 12.7 (MedCalc Software). In all analyses, a *P* < 0.05 was considered significant.

## Results

### Dataset

The clinical characteristics of the training and validation datasets are summarized in Table 1. The training dataset of 300 examinations for BA segmentation by Attention U-net included 150 patients in the normal PLC group (mean TLICS score, 1.78 ± 0.42; range, 1–2) and 150 in the injured PLC group (mean TLICS score, 4.22 ± 0.81; range, 2–8). The internal validation test of 100 examinations including 50 in the normal PLC group (mean TLICS score, 1.82 ± 0.39; range, 1–2) and 50 in the injured PLC group (mean TLICS score, 4.3 ± 0.80; range 3–7). The external validation dataset also consisted of 50 examinations with PLC injury (mean TLICS score, 1.59 ± 0.50; range 1–2) and 50 examinations without PLC injury (mean TLICS score, 4.29 ± 0.89; range, 3–7).

### Diagnostic performance of the model

#### First step result (segmentation result of DL) of attention U-net

The Dice similarity coefficient of attention U-net in the first step were 0.849 for BA and 0.919 for fractured vertebral body in the internal test set and 0.773 for BA and 0.853 for fractured vertebral body in the external test set (Table 2). The proportion of attention U-net segmented BA containing the area of manual segmented BA were 99% (99/100 examination) in the internal validation test and 95% (95/100 examination) in the external validation test.

**Table 2:** Performance evaluation of deep learning algorithm for internal and external validation datasets.

	Internal validation	External validation
First step (segmentation)		
DSC (BA/TL fracture)	0.849/0.919	0.773/0.853
Detection proportion of BA by Attention U-net segmentation	0.99 (99/100)	0.95 (95/100)
Second step (detection of the PLC injury)		
Sensitivity	0.880	0.820
Specificity	0.820	0.940
PPV	0.830	0.932
NPV	0.872	0.839
AUROC	0.928	0.916

^†^DSC, Dice similarity coefficient; BA, background anatomy; TL, thoracolumbar, PPV, positive predictive value; NPV, negative predictive value; AUROC, area under the curve of the receiver operating characteristics.

#### Second step result (detection of PLC injury) of inception-ResNet

The AUROC of the internal validation was 0.928. The sensitivity and specificity in the internal test set were 88.0% and 82.0%, respectively (Table 2). Six patients (two in the lumbar vertebral levels, one in the lower thoracic vertebral level, and three in the T12-L1 levels) with injured PLC were missed in the internal test set. The algorithm made nine false-positive detections in the internal test set; six had lumbar and three had thoracic vertebral fractures.

The AUROC of the external validation was 0.916. The sensitivity and specificity in the external test set were 82.0% and 94.0%, respectively. Nine patients (three in the lumbar, four in the lower thoracic, and two in the T12-L1 levels) with injured PLCs were missed in the external test set. The external test set had three false-positive detections of lumbar vertebral fractures (Table 2).

### Comparison result of the proposed DL system (two serial DL algorithms) with other DL algorithms

#### Comparison of first step (segmentation) result of traditional U-net vs. Attention U-net

For the first DL algorithm comparison, we used the traditional U-net to validate the effectiveness of attention gate. The Dice similarity coefficient of the TL fracture and BA of the two segmentation algorithms (traditional U-net / Attention U-net) were 0.789/0.853 and 0.697/0.773, respectively. In other words, the segmentation results of the TL fracture and BA by traditional U-Net are approximately 6.4% and 7.6% lower than those of Attention U-Net, respectively.

#### Comparison of second step (classification) result of inception-ResNet-V2 vs. other classification algorithms

The classification performance was verified by comparing several deep learning models other than Inception-ResNet-V2, which was the second DL algorithm in the proposed DL system. The DL algorithms used in the comparison were Inception-ResNet-V2 (proposed DL algorithm), InceptionV3^22^, Xception^23^, MobileNetV2^24^, and EfficientNetB7^25^, and the inputs used in this comparison process were all the same: the final image patches from the first (prepocessing of input image) and second processes (segmentation and patch extraction) of the proposed DL system. Additionally, in this process, the hyperparameters and data augmentation were set the same as the classification model of the proposed DL system. In terms of AUROC, the proposed model (Inception-ResNet-V2) performed the best with 0.916, followed by InceptionV3, EfficientNetB7, Xception, and MobileNetV2. In detail, the sensitivity was higher for InceptionV3 than for the proposed model. Nevertheless, InceptionV3 and other DL algorithms performed significantly worse than the proposed model on other diagnostic metrics, and the proposed model had the most stable performance when considering both normal and injured PLC classes (Table 3).

**Table 3:** Comparison of classification result of Inception-ResNet-V2 vs. other classification algorithms.

External validation dataset	InResNetV2 (Proposed)	InceptionV3	Xception	MobileNetV2	EfficientNetB7
Sensitivity	0.820	0.880	0.740	0.380	0.780
Specificity	0.940	0.680	0.760	0.840	0.680
PPV	0.932	0.733	0.755	0.704	0.709
NPV	0.839	0.850	0.745	0.575	0.756
AUROC	0.916	0.875	0.838	0.670	0.820

^†^ PPV, positive predictive value; NPV, negative predictive value; AUROC, area under the curve of the receiver operating characteristics;

InResNetV2, Inception-ResNet-V2.

#### Comparison of classification performance between the single algorithm (without segmentation and patch extraction) and the two serial DL algorithm (proposed DL system)

We obtained the performance of the classification model without the two processes, segmentation task and patch extraction, the second step of the proposed DL system, to validate the effectiveness of the two processes. That is, it is an experiment that performs preprocessing on the original MRI image, but omits the segmentation task and patch extraction task and directly uses the classification model to classify PLC injuries. The deep learning architecture for the classification comparison was Inception-ResNet-V2, InceptionV3, Xception, MobileNetV2, and EfficientNetB7, and the hyperparameters and data augmentation were set the same as the classification model of the proposed DL system. The classification performance of each model on the external validation dataset is presented in Table 4. The highest performing model was InceptionV3 with an AUROC of 0.730, followed by Inception-ResNet-V2, MobileNet-V2, Xception, and EfficientNetB7. Nevertheless, all models performed worse in AUROC than the proposed DL system predicted by the input after segmentation and patch extraction, and even InceptionV3, which performed the best among the single algorithm results, had an AUROC of 18.6% lower than the proposed DL system.

**Table 4:** Comparison of classification performance of Single algorithm result (single algorithm result) and the proposed DL system (two serial DL algorithm result).

External validation set	Proposed DL system	InResNetV2 (single-algorithm)	InceptionV3	Xception	MobileNetV2	EfficientNetB7
Sensitivity	0.820	0.340	0.500	0.200	0.420	0.280
Specificity	0.940	0.820	0.720	0.960	0.700	0.780
PPV	0.932	0.654	0.641	0.833	0.583	0.560
NPV	0.839	0.554	0.590	0.546	0.547	0.520
AUROC	0.916	0.624	0.730	0.505	0.536	0.498

^†^DL, deep learning; PPV, positive predictive value; NPV, negative predictive value; AUROC, area under the curve of the receiver operating characteristics;

InResNetV2, Inception-ResNet-V2.

### Comparison between the DL algorithm and radiologists

**Figure 4:**
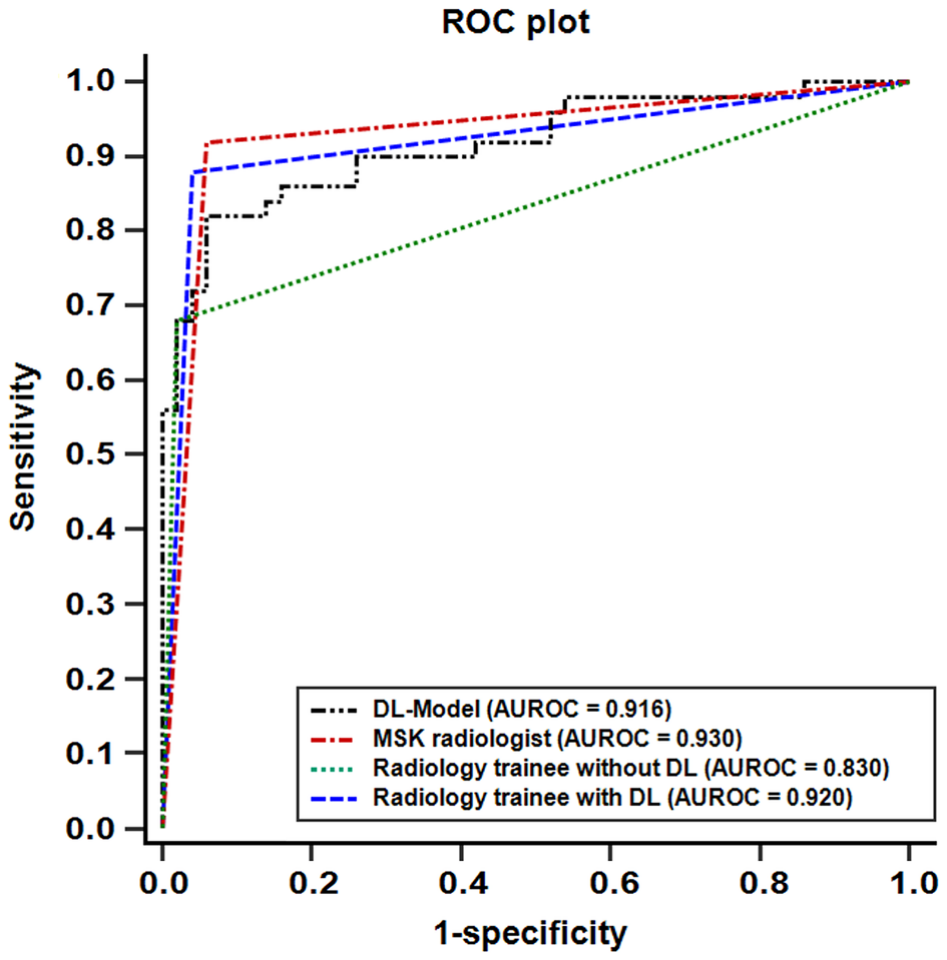
ROC plots for deep learning algorithm and radiologists in the external validation dataset. AUROC of the DL-algorithm was not significantly different from that of experienced musculoskeletal radiologist (R1, *p* = 0.722), but tended to be higher than that of radiology trainee (R2, *p* = 0.051) on Delong's test, close to statistical significance. There was significantly different in the AUROC between R1 and R2 (*p* = 0.011). In the second session with deep learning algorithm assistance, significant improvement in diagnostic performance was observed in R2 (increment of AUROC was 0.090, *p* = 0.007). (ROC, the receiver operating characteristics; AUROC, the area under the curve of the receiver operating characteristic; DL, deep learning).

In the first session of the observer performance test, the AUROCs for R1, and R2 were 0.930, and 0.830, respectively. There was no significant difference in the diagnostic performance of PLC injury between the DL algorithm and R1. However, the diagnostic performance of the DL algorithm tended to be higher (0.916) than that of R2 and was close to the statistically significant *p* value on Delong's test (*p* = 0.051). Furthermore, the diagnostic performance of R1 was higher than that of R2 (*p* = 0.011). The sensitivity and specificity of the two radiologists (R1/R2) were 92.0%/68.0% and 94.0%/98.0%, respectively. The accuracy of the two readers were 93.0% (R1) and 83.0% (R2). There was no significant difference between the algorithm and the two radiologists' diagnosis of PLC injury (*p* > 0.05). However, there was significant difference in the diagnosis of PLC injury between the R1 compared to R2 (*p* = 0.001). In the second session of observer performance test (R2 with DL-algorithm assistance), AUROC for R2 was 0.920. The increment of AUROC was 0.090 compared to the first session (*p* < 0.001). In terms of sensitivity, significant increment was shown (*p* = 0.006), while no significant difference of specificity (*p* > 0.999) was shown (Table 5 and Fig. 4).

**Table 5:** Performance of Deep learning and Radiologist in Diagnosing PLC injury.

	Sensitivity	Specificity	PPV	NPV	AUROC
DL algorithm	0.820 (0.686–0.914)	0.940 (0.835–0.988)	0.932 (0.819–0.976)	0.839 (0.742–0.905)	0.916 (0.844–0.963)
MSK radiologist (R1)	0.920 (0.808–0.978)	0.940 (0.835–0.988)	0.939 (0.836–0.979)	0.922 (0.821–0.968)	0.930 (0.861–0.971)
Radiology trainee (R2) without DL-assistance	0.680 (0.533–0.805)	0.980 (0.894–0.999)	0.971 (0.829–0.996)	0.754 (0.671–0.821)	0.830 (0.742–0.898)
R2 with DL-assistance	0.880 (0.757–0.955)	0.960 (0.863–0.995)	0.957 (0.849–0.989)	0.889 (0.790–0.944)	0.920 (0.848–0.965)

^†^Data in the parentheses are 95% confidence intervals.

^‡^PLC, posterior ligamentous complex; DL, deep learning; PPV, positive predictive value; NPV, negative predictive value; AUROC, area under the curve of the receiver operating characteristics; MSK, musculoskeletal.

### Grad-CAM

**Figure 5:**
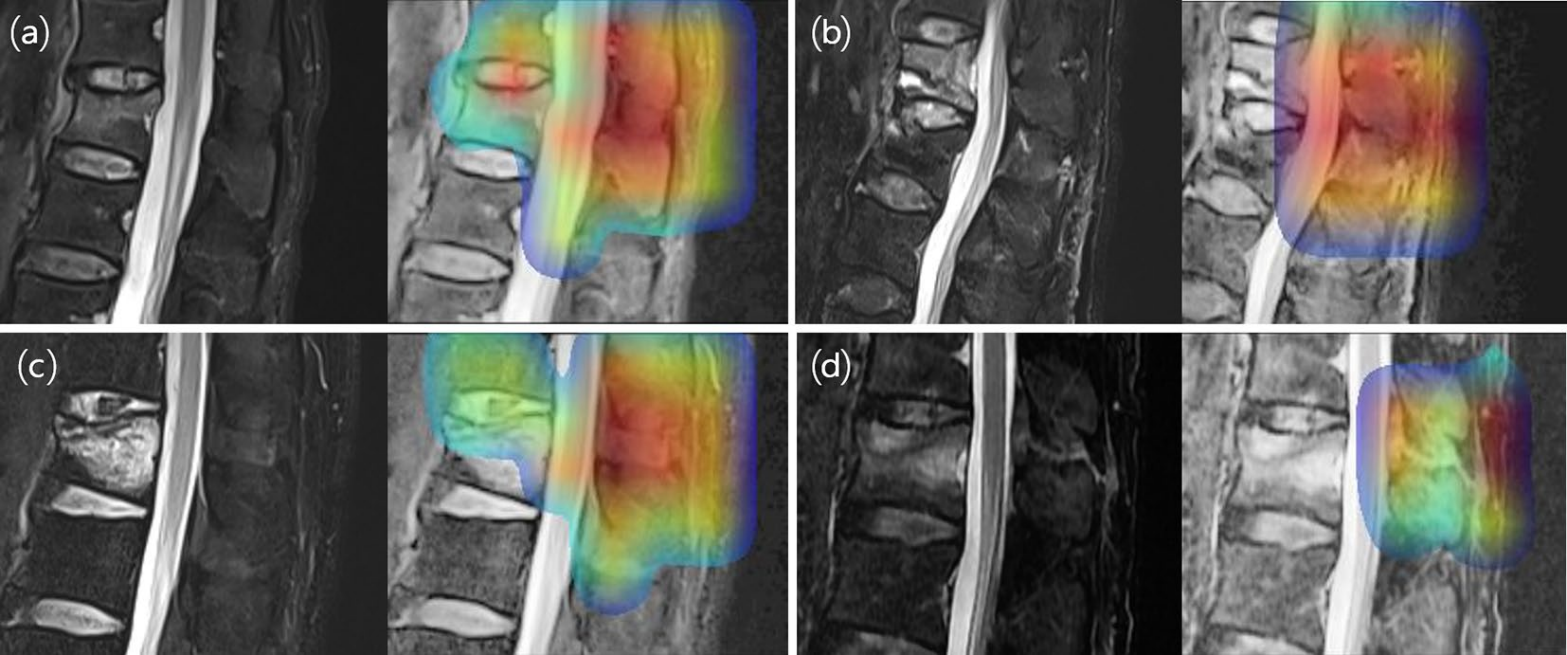
Representative four cases of FS sagittal T2 weighted image via gradient-weighted class activation mapping. **(a,c)** TL fracture cases with normal PLC. **(b,d)** TL fracture cased with injured PLC. Compared to injured PLC cases **(b,d)** and normal PLC cases **(a,c)**, it can be seen that Grad-CAM is more limited to the background soft tissue anatomy area including injured PLC. This can be interpreted as the DL algorithm making a judgment based on the area where the PLC is injured. (FS, fat suppression; TL, thoracolumbar; PLC, posterior ligamentous complex; DL, deep learning).

Grad-CAM was also used to provide a visual explanation of the decision and make it more transparent to better understand the functionality of the deep neural network^26^. Several examples of Grad-CAM map are illustrated in Fig. 5 for normal PLC and injured PLC. For the injured PLC, the Grad-CAM map was confined to the injured PLC and background anatomical regions. In the normal PLC group, the Grad-CAM map was more widely distributed than in the injured PLC case and included the BA, vertebral body, and spinal canal. This Grad-CAM map suggested that the deep neural network in the second step was properly trained and that the important areas for imaging diagnosis are being monitored by inception-ResNet-V2.

**Figure 6:**
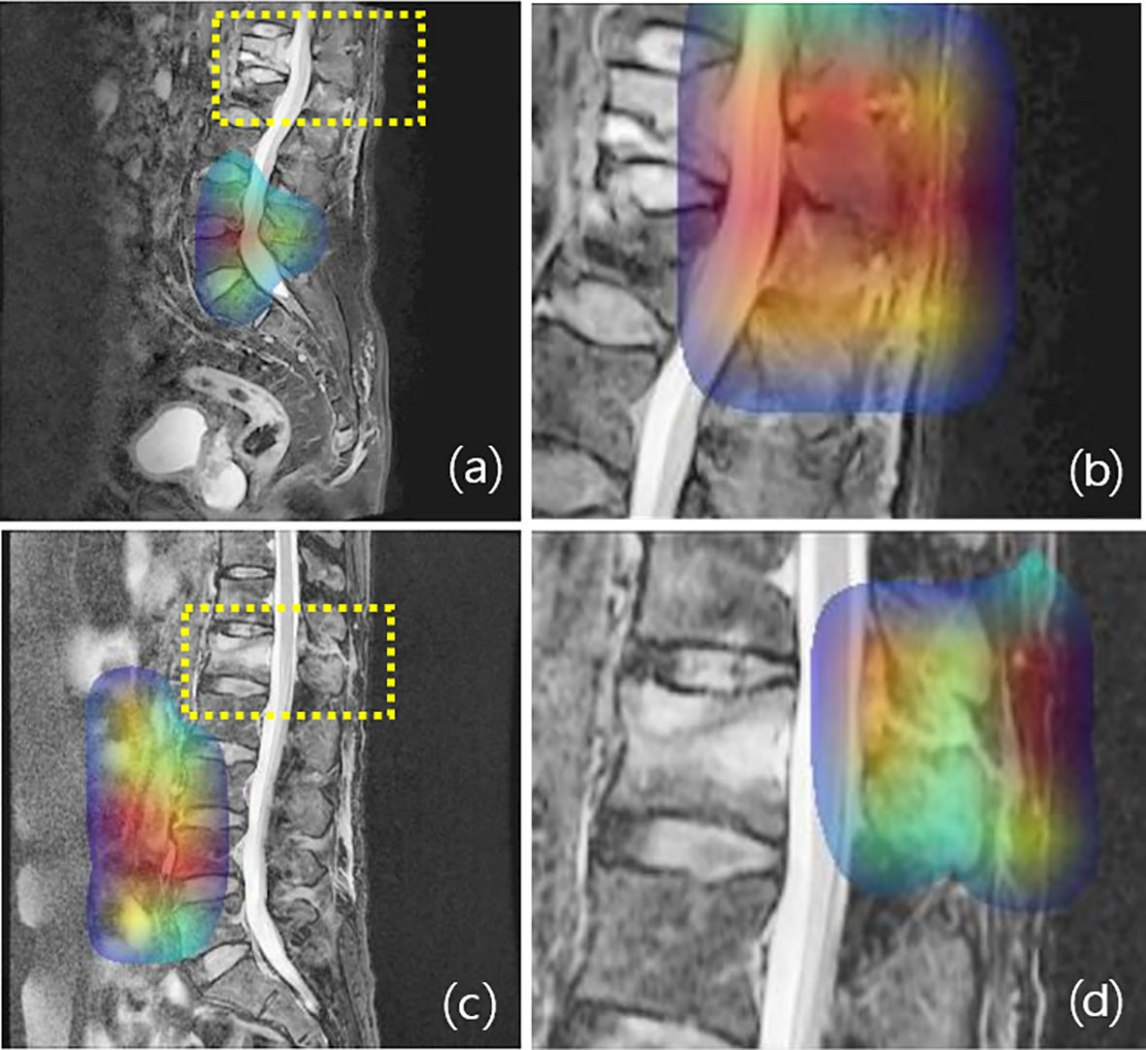
Comparison of Grad-CAM results for a single classification algorithm (left column) and the proposed DL system (right column). (**a**) and (**c**) are FS T2 sagittal images of two different TL fracture patients with PLC injury. The GRAD-CAM results for Single classification algorithm are (**a**) and (**c**), and the GRAD-CAM results for the proposed DL system of (**a**) and (**c**) images are (**b**) and (**d**), respectively. The yellow dashed boxes indicates the final image patches resulting from the segmentation task and patch extraction. As shown in the figure, the single classification algorithm (**a**,**c**) makes predictions based on different locations than the final image patches extracted by patch extraction, such as the yellow boxes, unlike the proposed DL system (**b**,**d**). This disparity explains why the AUROC of the single classification algorithm is much lower than that of the proposed DL system (FS, fat suppression; TL, thoracolumbar; DL, deep learning).

In some cases, the classification algorithm in the second step was not affected by the BA segmentation (attention U-net) in the first step (Supplementary Fig. 2), while in other cases it was highly affected (Fig. 6). The inception-ResNet-V2 algorithm detected one injured PLC from one failed BA segmentation in the PLC injury group of the internal test set. Furthermore, the DL algorithm accurately classified one normal and three injured PLC cases from five (one normal and four injured PLC cases) failed BA segmentation cases in the external test set (Supplementary Fig. 2).

In the comparison of classification performance without segmentation and patch extraction and the proposed DL system, the single classification algorithm performed poorly compared to the proposed DL system (two serial DL algorithms), and to identify the reason for this, Grad-CAM was used to determine which part of the image was utilized by the DL algorithms to predict classification. As a result, unlike the proposed DL system, there were cases where the single classification algorithm failed to focus on the fractured vertebral body, which occupied a small area of the entire sagittal image (Fig. 6). Therefore, we inferred that the AUROC was relatively low because the single classification algorithm often based its predictions on locations unrelated to the TL fracture and PLC injury.

## Discussion

This study is the first study on MRI-based PLC injury in TL fractures. Two-step training of the DL system was effective for the detection and classification of PLC injury showing AUROCs of 0.927 and 0.916 in the internal and external validation sets, respectively, which was similar to the performance of MSK radiologists. And we also revealed improvement in radiologist performance with the aid of the DL algorithms.

The reasons for this high diagnostic performance can be speculated as follows. First, in this study, BA including the PLC was segmented using Attention U-net, and the image range for finding features was dramatically reduced by patch extraction only around the segmentation. As shown in the single classification algorithm results, without the segmentation and patch extraction process in the first step, the performance was significantly reduced because the corresponding feature had to be detected at all spine levels included in the image. Therefore, the area that the DL algorithm should evaluate was reduced to BA above and below the vertebral body fracture, and then the features were extracted. Through the comparison of classification performance between the single algorithm and the two serial DL algorithms, we clearly confirmed that the first step significantly improved the performance of Inception-ResNet-V2 in the second step. Second, in the first step, the Attention U-net showed higher accuracy in terms of the Dice similarity coefficient compared to the traditional U-net. A previous study reported the attention gates in Attention U-net improve the sensitivity and accuracy for dense label predictions by suppressing feature activations in irrelevant regions^20^. Another study on lumbar spine MRI reported that the use of Attention U-net increased the accuracy of lumbar spine segmentation^27^. Similar to previous studies, our study showed that the fractured vertebral body and the BA segmentation results of the Attention U-net were superior to those of the traditional U-net. Third, the Inception-ResNet-V2 used in the second step is an algorithm that combines residual blocks with Inception previously developed by Google^21,22,28,29^. Due to this combination, the amount of memory and computation almost doubled compared to Inception V3, but it combined the advantages of ResNet, 'Compensating for the problem (Gradient Vanishing/Exploding, an increase in error due to an increase in the number of parameters) that occurs when the network is deep with residual connections'; thus, it has higher performance than the general Inception algorithm^21^. In our study, similar to previous studies, Inception-ResNet-V2 performed better in terms of AUROC compared to Inception V3. Inception-ResNet-V2 has been reported as a deep neural network with high accuracy in medical image classification^30,31^, and our study also showed high accuracy despite relatively few training cases.

The comparison between Inception-ResNet-V2 and other classification algorithms showed that Inception-ResNet-V2 had better diagnostic performance in terms of AUROC than other classification algorithms. Due to this, we selected Inception-ResNet-V2 as the backbone model for the classification task (second step). Notably, Efficient NetB7 had a slightly lower AUROC value than Inception V3 and Inception-ResNet-V2, despite being a more recently developed DL algorithm. The exact reason for this is unclear, but it is possible that the relatively small training data set in this study made it difficult to make accurate comparisons between models. Also, for DL algorithms with relatively complex compound scaling methods such as Efficient NetB7^25^, it is possible that applying the same hyperparameters and data augmentation methods as applied to the Inception V3, Inception-ResNet-V2 algorithms may not be the most optimal method. This may require further research using more training data in the future study.

The algorithm developed in this study was also evaluated using an external dataset of images taken using different equipment from other MRI vendors in different hospitals. In the external validation dataset, unlike the internal dataset, the injured PLC group had a different gender distribution and mean age. Additionally, in the external dataset, the injured PLC group had more cases with two vertebral body fractures than one, compared to the injured PLC group in the internal dataset. This external validation suggested that the algorithm might be robust and effective for general use in real clinical settings.

The importance of PLC evaluation in TL fracture is well known, and the presence or absence of PLC injury is essential in surgical decision-making due to its effect on the stability of the fracture site^1,2,32^. Evaluation of PLC is mostly performed using MRI, and in most fracture patients, additional MRI is performed for PLC injury or neurological evaluation^4,5,9^. In determining surgical treatment, TLICS is commonly used by many clinicians because it reflects the spinal stability and the biomechanical mechanism of the spine^4,33^. The MRI-based evaluation of PLC injury depends more on the radiologist than on the evaluation of fracture morphology. It can be more difficult for a surgeon to assess a PLC injury using MRI than evaluate the TL fractures. Therefore, the role of the radiologist is even more important in the MRI-based evaluation of PLC injury. Inter- and intraobserver agreement on PLC injury was fair to moderate (0.389–0.616) among radiologists using MRI; radiologists possibly tend to over-recognize PLC conditions on MRI^34^ possibly because the subjective evaluation of MRI-based PLC injury and opinions may differ between surgeons and radiologists. Thus, in this context, the DL system proposed in our study has the potential to help radiologists in the consistent diagnosis of PLC injuries and surgeons in the evaluation of PLC injuries.

Until recently, studies using the DL system on TL spine fractures covered radiography and CT^10,14,17,35,36^. However, few studies on TL spine fracture using CT or MRI have been conducted. DL studies using MRI are limited because diagnosis is complicated and data collection is difficult because the segmentation of the anatomical structure of the spine must be the basis and fractures need to be diagnosed using multiplane imaging data. In particular, the PLC contains several ligamentous structures that complicate evaluation; most PLC injuries are in the interspinous or supraspinous ligament, and the most visible location is the spinous process, which is midline and visible on the sagittal plane. Therefore, in this study, the complicated process of data collection could be further simplified because the evaluation of the PLC was possible even if only one image was evaluated.

This study has some limitations. First, a small number of training datasets were used. In DL development, more accurate and sophisticated training is possible with more data. However, in our study, we were able to increase the diagnostic accuracy even though we used a small sample by using a two-step DL system. Second, PLC was evaluated with only one mid-sagittal image of MRI. However, most of the injuries were of the interspinous and supraspinous ligaments; in the absence of skipping in the mid-sagittal image, PLC evaluation was sufficient with only one image. Although there are limitations in assessing injuries to only the facet joint capsule or ligamentum flavum, these cases are extremely rare. Finally, false positives in the PLC evaluation are likely, such as due to interspinous bursitis at the lower lumbar level. However, considering that findings of interspinous bursitis also lead to disagreements in the diagnosis of PLC injury even among radiologists, this may be an inherent limitation of imaging diagnosis of PLC injury.

In conclusion, the DL algorithm detected PLC injury in patients with TL fracture with a high diagnostic performance, which was comparable to that of an experienced MSK radiologist. Therefore, PLC evaluation using the proposed DL algorithm may benefit radiologists and surgeons by enabling efficient and accurate screening of PLC injuries.

### Supplementary Information

Supplementary Information.

## Data Availability

All data generated or analyzed during this study are included in this published article (and its Supplementary Information files).
