# Supplementary material for: Deep learning system for automated detection of posterior ligamentous complex injury in patients with thoracolumbar fracture on MRI
Source: Sci Rep. 2023 Nov 3;13:19017. doi: 10.1038/s41598-023-46208-7 (PMC10624679; doi:10.1038/s41598-023-46208-7)

**Supplementary Information**

**Supplementary Method 1.**

***Subjects***

Between January 2019 to December 2021, the image database contained 583 consecutive patients who underwent baseline thoracic or lumbar spine MRI after trauma and were diagnosed to have one or two continuous acute TL fractures. Those who met the following criteria were excluded from the training and internal validation : 1) Evaluation was limited due to poor quality of MRI or artifact due to previous surgery or procedure (n=89), 2) No fracture in the TL vertebral body (n=27) (e.g., an isolated PLC injury, transverse or spinous process fracture, or sacral fracture only), 3) High signal changes due to any etiology other than trauma in a vertebral body other than the fractured one (e.g., a pathologic fracture, infection, inflammation, or benign bone tumor such as hemangioma) (n=16), 4) Vertebral deformity such as severe scoliosis (Cobb angle >20) (n=8), 5) Equivocal findings of injured PLC about which two radiologists did not agree in diagnosing PLC injury, especially if combined with confounders such as interspinous bursitis or non-specific subcutaneous back edema. (n=43). Eventually, 400 patients with acute TL fractures were included. This was randomly divided into two: a training data set of 300 patients (150 with PLC injury and another 150 with normal PLC) and an internal validation set of 100 patients (50 with PLC injury and another 50 with normal PLC).

We added a dataset from another institution for external validation. Unlike the internal validation dataset, the external validation dataset consisted of different MRI scanners from different vendors. We extracted patients with acute TL fractures, 56 with PLC injury and 59 without PLC injury, from the radiologic database of another institution from January 2018 through February 2019. All personal information of patients was anonymized. The exclusion criteria of both external and internal validation datasets were identical, and 15 patients were excluded. Ultimately, the external validation set consisted of 50 TL fracture patients with PLC injury and another 50 TL fracture patients with normal PLC.

**Supplementary Table 1.** Spine MRI parameters in our institution (Skyra 3.0T, Siemens, Erlangen, Germany)

| **Species of MRI scan** | **T-spine MRI** | | | | | **L-spine MRI** | | | | |
| --- | --- | --- | --- | --- | --- | --- | --- | --- | --- | --- |
| Parameter | Sagittal | Sagittal | Sagittal | Axial | Axial | Sagittal | Sagittal | Sagittal | Axial | Axial |
|  | T2-WI TSE | T1-WI TSE | T2-WI TSE FS | T2-WI TSE | T1-WI TSE | T2-WI TSE | T1-WI TSE | T2-WI TSE FS | T2-WI TSE | T1-WI TSE |
| TR (msec) | 2650~3000 | 390~785 | 3000~4340 | 3000~6310 | 439~785 | 2860~4410 | 416~567 | 3000~4340 | 3600~7710 | 418 ~ 788 |
| TE (msec) | 81~83 | 10 | 70~82 | 100 | 10~11 | 82 ~ 90 | 10 | 84 | 100 | 15 |
| Matrix size | 448x314 512x410 | 448x314 512x307 | 384x230 384x307 | 384x230 | 320x208 384x230 | 512x410 | 448x269 | 448x314 | 384x230 | 384x230 |
| FOV (cm) | 32x32 34x34 | 32x32 34x34 | 32x32 34x34 | 16x16 | 16x16 | 34x34 | 34x34 | 34x34 | 16x16 | 16x16 |
| Section thickness (mm) | 3 | 3 | 3 | 4~6 | 4~6 | 3 | 3 | 3 | 4 | 4 |
| Intersection gap (mm) | 0.6 | 0.6 | 0.6 | 0.4~1.1 | 0.4~1.1 | 0.2 | 0.2 | 0.2 | 0.1 | 0.1 |
| Echo train length | 15 | 3 | 15~17 | 15 | 4 | 19 | 5 | 17 | 16 | 3 |
| No. of signals acquired | 2 | 1 | 2 | 2 | 2 | 2 | 1 | 2 | 2 | 2 |
| scan time (s) | 93~122 | 112~170 | 135~144 | 155~167 | 123~134 | 110~145 | 90~101 | 171~221 | 137~194 | 147~199 |

† TE, echo time; TR, repetition time; No, number; FOV, field of view; WI, weighted image; TSE, turbo spin-echo; FS = fat saturation

**Supplementary Table 2.** Spine MRI parameters in external institution (Signa HDxt 1.5T, GE Healthcare, Milwaukee, Wis, USA)

| **Species of MRI scan** | **T-spine MRI** | | | | | **L-spine MRI** | | | | |
| --- | --- | --- | --- | --- | --- | --- | --- | --- | --- | --- |
| Parameter | Sagittal | Sagittal | Sagittal | Axial | Axial | Sagittal | Sagittal | Sagittal | Axial | Axial |
|  | T2-WI TSE | T1-WI TSE | T2-WI TSE FS | T2-WI TSE | T1-WI TSE | T2-WI TSE | T1-WI TSE | T2-WI TSE FS | T2-WI TSE | T1-WI TSE |
| TR (msec) | 3566~4016 | 716~717 | 3983~5816 | 3216~4866 | 533~816 | 2633~3800 | 416~715 | 3000~3800 | 2833~9170 | 418~546 |
| TE (msec) | 73~92 | 8~9.6 | 81~93 | 80~90 | 8~10 | 73~92 | 9.5-10 | 73~84 | 90~102 | 11~15 |
| Matrix size | 448x224 | 384x224 | 448x224 | 320x224 | 320x192 | 384x224 | 384x224 | 448x314 | 320x224 | 320x224 |
| FOV (cm) | 34x34 | 34x34 | 34x34 | 18x18 | 18x18 | 28x28 | 28x28 | 34x34 35x35 | 18x18 | 18x18 |
| Section thickness (mm) | 3.5 | 3.5 | 3.5 | 3 | 3 | 4 | 4 | 4 | 3 | 3 |
| Intersection gap (mm) | 0.1 | 0.1 | 0.1 | 1 | 1 | 0.1 | 0.1 | 0.1 | 1 | 1 |
| Echo train length | 25 | 4 | 25 | 16 | 4 | 16~21 | 4 | 17~20 | 16 | 4 |
| No. of signals acquired | 2 | 1.5 | 2 | 2 | 1.5 | 2 | 1.5 | 2 | 2 | 1.5 |
| scan time (s) | 160-200 | 124-142 | 150-170 | 227-240 | 182-200 | 124-147 | 110-144 | 172-210 | 140-203 | 160-208 |

† TE, echo time; TR, repetition time; No, number; FOV, field of view; WI, weighted image; TSE, turbo spin-echo; FS = fat saturation

**Supplementary Figure 1.** Representative case of manual segmentation of TL fracture, background soft tissue anatomy, and injured PLC. The blue, yellow, and orange color areas represent TL vertebral fracture, background soft tissue anatomy, and injured PLC, respectively. (TL, thoracolumbar; PLC, posterior ligamentous complex)


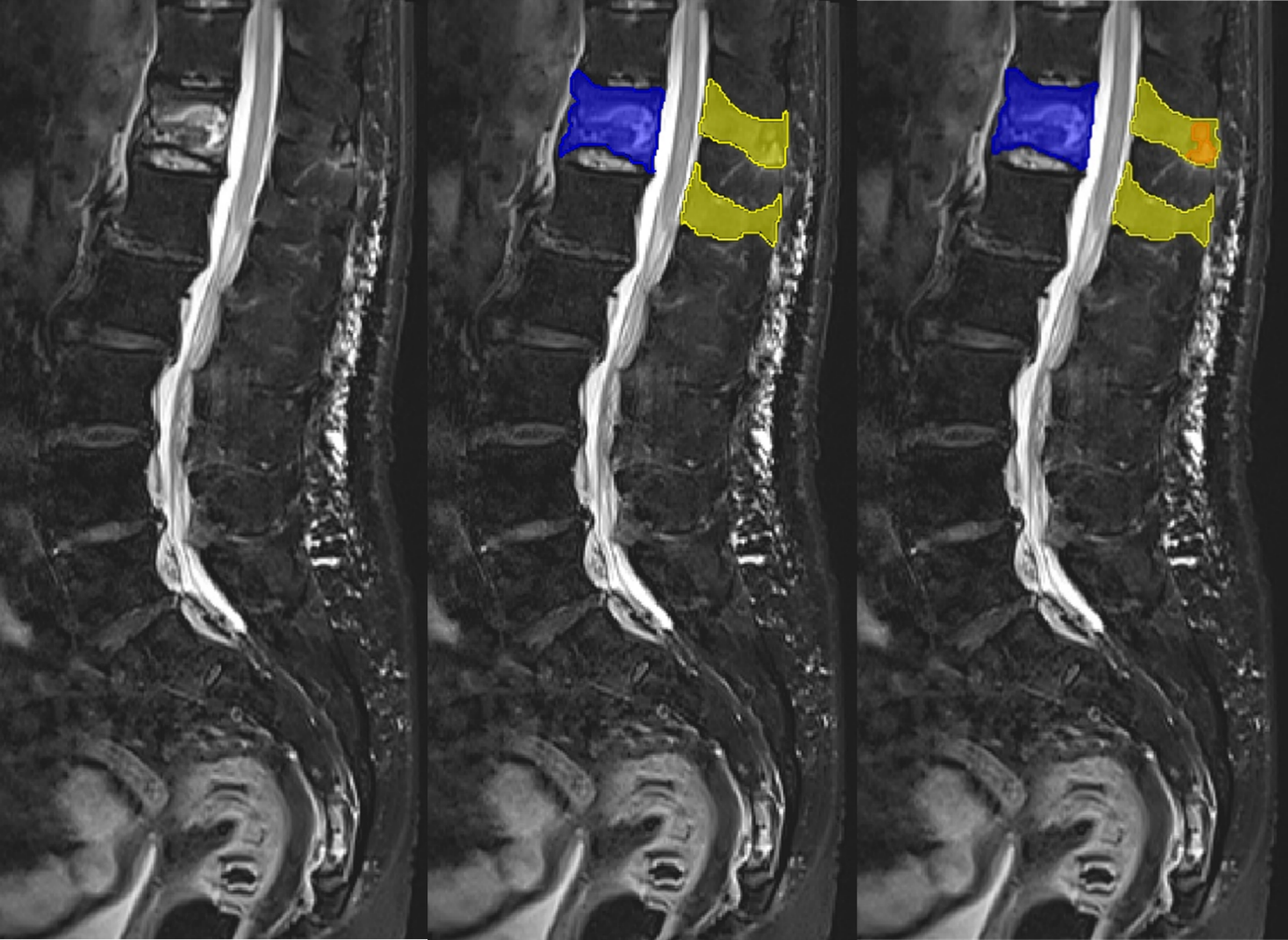


**Supplementary Figure 2**. Detection of injured PLC in the second step in cases of segmentation failure in the first step. The upper row pictures **(a-c)** are thoracic vertebral fracture case with PLC injury of internal validation dataset, and the lower row pictures **(d-f)** are lumbar vertebral fracture case with PLC injury of external validation dataset. The left column pictures **(a, d)** are images overlayed on DICOM with manual segmentation, respectively. Blue represents vertebral body fracture segmentation, and white represents background soft tissue anatomy segmentation. The middle row pictures **(b, e)** are images in which green color appears if the segmentation of the Attention U-net overlaps the manual segmentation area. As shown in the picture, there is no green color in the middle row picture, and only red color, which is manual segmentation, is shown, indicating that Attenuation U-net failed in segmentation. As shown in the gradient-weighted class activation map of the right column pictures **(c, f)**, even though a relatively large area was judged from the entire image without patch extraction, the Inception ResNet-V2 algorithm was able to detect the presence of PLC injury. (PLC, posterior ligamentous complex)


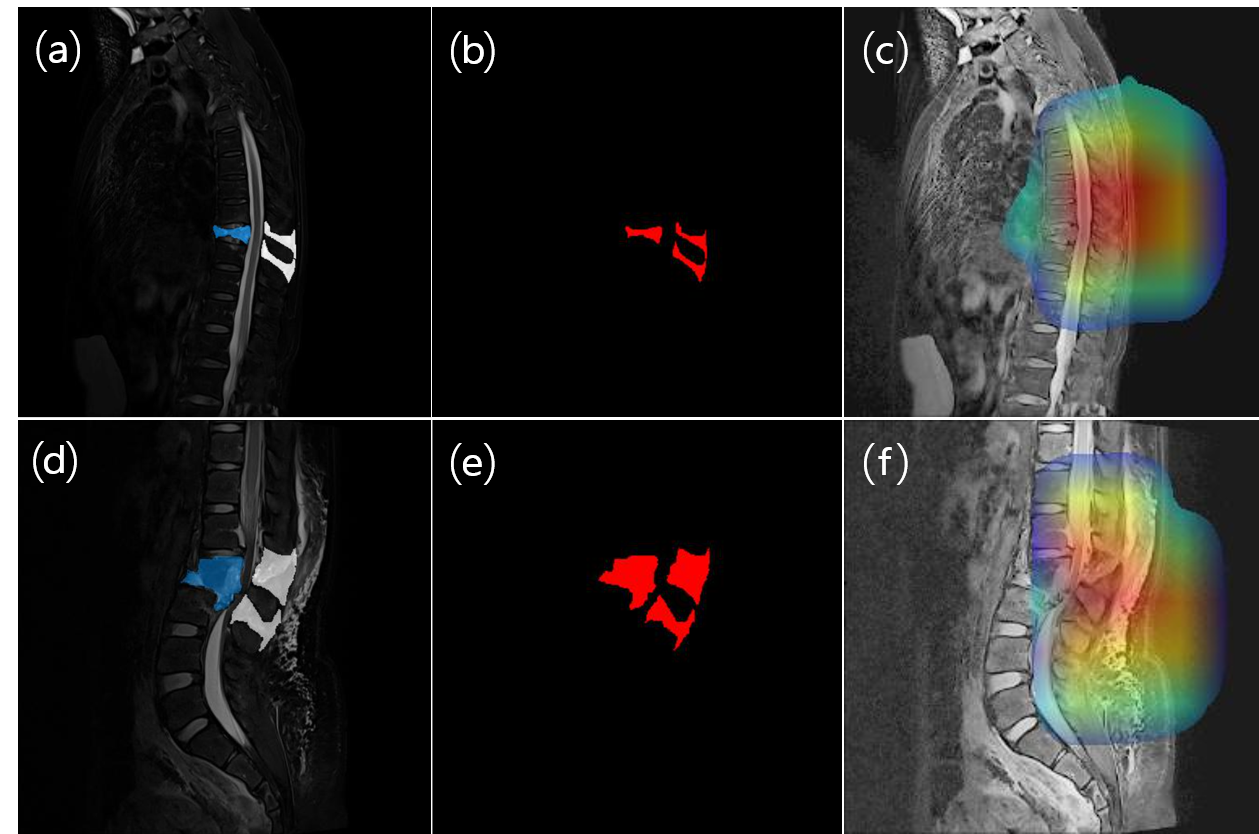

Supplement: Supplementary file 1 — Supplementary Information. [file 41598_2023_46208_MOESM1_ESM.docx]
